# The Final Report of the Committee of Inquiry

**Published:** 1917-02-24

**Authors:** 


### NATIONAL INSURANCE ACT DEVELOPMENTS.

### The Final Report of the Committee of Inquiry.

THE Departmental Committee of Inquiry, appointed by the Treasury in January 1916 to consider and report upon the financial scheme of the National Insurance Acts and the simplification of the administration of the approved societies, have recently completed their labours by the issue of their Final Report (Cd. 8451, 3d. net). We have already commented in some detail in THE HOSPITAL on the interim and further Reports of the Committee, which dealt respectively with the general financial scheme of the Acts, and the simplification of the practical machinery by which the benefits are administered. These Reports, valuable as they are and must be to those engaged in the business of national health insurance, are essentially technical in character, and do not, therefore, make any strong appeal to those who are untrained in the niceties of financial adjustment of receipts and expenditure, or to those who have not had any practical experience of the collection of the thousands of insurance cards and the payment of benefits, under cumbrous regulations and in an almost endless variety of conditions, which go to make up the daily work of the officials of approved societies.

It is, therefore, with a sense of relief, almost amounting to pleasure, that one is able to turn to the Final Report, and find therein matter of such character as to be generally appreciated and understood by those who have not had the advantage of the severe training in the complicated detail to which reference has been made.

In spite of all the multifarious activities of the National Health Insurance authorities, ranging from the Commissioners themselves to the humblest representative of the approved societies, there are still provisions of the Act of 1911 which have not been put into effective operation. The design of the authors of that Act was as much to prevent the occurrence of sickness as to cure sickness already contracted, and the title of the measure gave due prominence by the use of the words "prevention and cure" to the double object which its provisions were intended to serve.

Little has yet been done, however, towards the attainment of their double ideal. Almost necessarily, perhaps, owing to the practical measure in pounds, shillings, and pence of contributions on the one hand and of benefits on the other, attention became focussed at the outset upon the regulations necessary to secure that no one, fulfilling the description of an employed person within the meaning of the Act, escaped the meshes of the scheme. Having effectively secured the millions of insured persons the authorities have found their attention retained by the intricate problems of fitting the scheme to the endless variety of cases which have to be dealt with individually, and little time or opportunity has been afforded for enlarging the scope of National Insurance in the direction of the prevention of sickness which was originally contemplated, and for which, in some measure, provision was made.

PREVENTION OF SICKNESS.

It is to this aspect of the National Insurance Acts that the Committee of Inquiry direct attention in their Final Report, and whatever may be done in the way of financial readjustment or in the matter of simplifying administrative machinery, it is to be hoped that the strong recommendations of the Committee as to the wider application of the powers already conferred by the Acts, and for a more comprehensive interpretation of the intention of those Acts, will be put into operation. The common-sense point of view, which is so strongly indicated in some of the recommendations, gives evidence of the wide knowledge of human nature possessed by the Committee, and of the acumen and foresight which mark those who are truly successful business organisers. Thus, for instance, they recommend the payment of sickness benefit to persons who would, in existing circumstances, be debarred from its receipt under the misconduct rule, not with any idea of condoning the misconduct, but with the object of effecting a speedy recovery of the sufferer, thus ensuring the double advantage of preventing the spread of the fell disease to those who are innocent, and of a direct saving in the payment of benefits, possibly for an extended period and of an expensive character, which would almost certainly result if the early cure were neglected.

This is the broad view which, though it may involve additional immediate expenditure, will more than justify such expenditure by ultimate saving, and will in addition secure to the nation rehabilitated workers and provide a direct benefit to the general health of the community.

The recommendations of the Committee in their Final Report are considered under three main headings—namely (1) matters relating to inquiries into causes of excessive sickness, (2) the payment of benefits in sickness due to venereal disease, and (3) the payment of benefits in the case of tubercular disease during the period of after-care. As the recommendations are all of considerable interest, we proceed to summarise them in the order in which they are dealt with in the Report.

### Excessive Sickness.

Section 63 of the Act of 1911 was provided to afford the means for ascertaining if excessive sickness among insured persons was due to the conditions or nature of the employment of such persons, or to bad housing or insanitary conditions in any locality, or to an insufficient or contaminated water supply, or to neglect to observe or enforce provisions of any public health Act or public health precautions, and to enable the approved societies and Insurance Committees to recover the cost of the benefits paid on account of such excess sickness from the person or authority through whose fault or neglect the excessive sickness resulted.

This section has not been utilised heretofore on account of the difficulty of applying the standard laid down for the calculation of the excessive sickness, and of the onus which it placed upon approved societies of initiating proceedings, without being able to obtain in advance any certain indication as to the success or otherwise of the result of the contemplated action. Moreover, the Committee point out that the standard addition of 10 per cent. to the normal sickness rates, which must be proved to have been exceeded before a claim for compensation could be made effectively under the section, could not be applied with fairness to all classes alike. Certain occupations are from their inherent nature more deleterious to health than others, and thus a fixed standard applicable to all alike would be inequitable.

In these circumstances the Committee suggest that the fixed standard should be abandoned, and that upon an inquiry being made the person holding the same should be vested with full discretion to assess the loss sustained by a society on the facts placed before him, without reference to any pre-determined standard. It is also suggested that the procedure for the institution of an inquiry should be amended, by making it necessary for application to be made in the first instance to the Insurance Commissioners, and that not until a *prima facie* case has been established to their satisfaction is any action to be taken against the person or authority through whose alleged fault or neglect the excessive sickness has occurred. The Committee also recommend that facilities should be provided for joint action where several societies operating in the same area are affected.

From the point of view of prevention of excessive sickness nothing could be more effective than to bring home the fact of such excess to those who are the cause thereof, and to require from them the payment of the loss which has been occasioned thereby to approved societies. If only a few well-chosen examples were selected and brought to a successful conclusion, it is safe to say that conditions would not only be altered in the particular instances selected, but the far-reaching effect would be of inestimable benefit to public health in general.

### Payment of Benefit in Sickness due to Venereal Disease.

The general recommendations of the Committee under this heading have already been briefly referred to in the introductory remarks above. It may, however, be mentioned that the Committee had the advantage of hearing witnesses representing the National Council for Combating Venereal Diseases, the British Medical Association, and the Panel Medico-Political Union. The Memoranda submitted by these expert bodies are incorporated in appendices to the Report, and they are unanimous in their plea for the assistance of the machinery of National Insurance as an aid in the suppression of the curse.

Following the practice of the old Friendly Societies, the model rules, put forward for adoption by approved societies by the Insurance Commissioners, incorporated a rule against the payment of sickness and disablement benefits when the sickness was due to the personal misconduct of the member. In most cases this has been interpreted as excluding from benefit any person suffering from venereal disease. But the Committee point out that there are many innocent sufferers, and if there were to be any effective discrimination between the innocent and the guilty there would of necessity be involved delicate and difficult inquiries.

The Committee indicate that it will not be necessary to introduce special legislation to give effect to their recommendation that venereal disease shall not of itself debar the sufferer from receiving the benefits, but they state that some remodelling of the rules of societies may be necessary to govern the question of conduct during sickness. It may be said, briefly, that their recommendation is that benefit shall only be allowed provided the sufferer submits to and follows out the course of treatment recommended by the doctor. We understand that several of the more important approved societies have already altered their rules and practice in conformity with these recommendations, and it is to be hoped that all societies, from the greatest to the least, will adopt them as soon as possible.

### Payment of Benefits to Tuberculous Patients during After-Care.

Witnesses, who were specially qualified to speak on the matter, appeared before the Committee and laid emphasis on the contention that a patient's treatment should not cease when he is discharged from a sanatorium, but that suitable treatment should be continued for a considerable period until he is able to return to full employment. During this interval, which is a critical period of his treatment, he is often unfitted to do a full day's work; at the same time he may not be incapable of doing any work whatever. The question therefore arises whether it is possible in the circumstances for sickness benefit to be paid when, according to the fundamental definition of that benefit, it is necessary for the insured to be incapable of work. Two schemes were submitted to the notice of the Committee, both of which are incorporated in appendices to the Report, and they comment favourably upon them. The idea, briefly, is that if an insured person is engaged at an approved institution for the after-care of tuberculous patients, even though he may be able to earn some small sums by his labour, he shall not be debarred from the receipt of benefit. If, however, the patient has returned to his own home and is not an inmate of an approved institution the Committee see graver difficulties in the extension of the right to benefits. At the same time they suggest that the difficulty might in part be overcome if the relief were given at the discretion of some outside association, specially set up for the purpose, and divorced from the normal administration of approved societies. It would be possible for such associations to be supported by subscriptions or donations from approved societies, under the existing provisions of the Act which allow such contributions to charitable institutions to be treated as expenditure on benefits.

